# “Joy of life” in nursing homes. Healthcare personnel experiences of the implementation of the national strategy. A qualitative study with content analysis of interviews

**DOI:** 10.1186/s12913-021-06801-w

**Published:** 2021-08-04

**Authors:** Beate André, Kjersti Grønning, Frode F. Jacobsen, Gørill Haugan

**Affiliations:** 1 Department of Public Health and Nursing, Norwegian University of Science and Technology (NTNU), 7491 Trondheim, Norway; 2 NTNU Center for Health Promotion Research, 7491 Trondheim, Norway; 3 Centre for Care Research, Western Norway University of Applied Sciences, Bergen, Norway; 4 Institute for Health and Care Sciences, Western Norway University of Applied Sciences, Bergen, Norway; 5 VID Specialized University, Bergen, Norway

**Keywords:** Implementation, Nursing homes, Healthcare personnel, Joy-of-life strategy, Qualitative interview study, Content analysis

## Abstract

**Background:**

Nursing homes are under strong pressure to provide good care to the residents. In Norway, municipalities have applied the ‘Joy-of-Life-Nursing-Home’ strategy to increase a health-promoting perception that focuses on the older persons` resources. Implementations represent introducing changes to the healthcare personnel; however, changing one’s working approaches, routines and working culture may be demanding. On this background, we explored how the ‘Joy-of-Life-Nursing-Home’ strategy is perceived by the employees in retrospective, over a period after the implementation and which challenges the employees experience with this implementation.

**Method:**

We used a qualitative approach and interviewed 14 healthcare personnel working in nursing homes in one Norwegian municipality, which had implemented the ‘Joy-of-Life-Nursing-Home’ strategy. The analysis was conducted following Kvale’s approach to qualitative content analysis.

**Results:**

The main categories were: (a) the characteristics of care activities before implementations of ‘Joy-of-Life-Nursing-Home’, (b) how ‘Joy-of-Life-Nursing-Home’ influenced the care activities, and (c) challenges with the implementation of ‘Joy-of-Life-Nursing-Home’. Some of the informants spoke well about the implementation concerning the care quality stating “*to see the joy in the eyes of the resident then I feel we have succeeded”.* For informants who experienced resistance toward the implementation, they felt it was too much to document, it was too complicated, and the requirements were too many.

**Conclusions:**

Quality of care seems to have increased after the implementation, as perceived by the informants. Nevertheless, the fact that the informants seemed to be divided into two different groups related to their main perspective of the implementation is concerning. One group has positive experiences with the implementations process and the benefits of it, while the other group focuses on lack of benefits and problems with the implementation process. To understand what facilitates and hinders the implementation, research on contextual factors like work environment and leadership is recommended.

## Contributions to the literature

- Healthcare personnel experienced increased quality of care for the residents after the implementation of the Joy-of-life strategy.
- It seems that the healthcare professionals are more conscious of their own behavior and attitudes after the implementation.
- Making changes to increase care quality in nursing homes is positive both for the residents and for the healthcare personnel; one way to succeed seems to be to implement the Joy-of-life strategy.

## Background

Healthcare personnel working in nursing homes (NH) are under strong pressure to provide good care to the residents [1, 2]. It is difficult to recruit and retain qualified workers [3, 4]. The focus on professional development and attitudes is therefore of great importance for NHs to appear as an attractive workplace for health professionals [1]. Eldercare concept positions that municipalities need to poise properly their eldercare innovations into three key groups; improving the quality of care for the elderly, improving the working environment, and societal efficiency [5].

Currently, about 40.000 people live in Norwegian NHs [6]. To facilitate wellbeing and quality of life (QoL) among NH residents’ and their families, the “Joy-of-Life-Nursing-Home” (JoLNH) strategy was established in Norway. The JoLNH is a national strategy for endorsing comfort, meaning-in-life and QoL among NH residents [6]. Several Norwegian municipalities have applied the JoLNH strategy which is founded on a health-promoting perception focusing on the older persons` resources and own interests and goals. The Norwegian government strongly endorses introduction of the JoLNH authorization to the municipal health services [6]. To convert into a qualified JoLNH, the NH must accomplish nine standards established by the Joy of Life (JoL) foundation: 1) all staff must be familiar with the JoLNH philosophy and its implications, 2) the NH must facilitate cooperation with schools, kindergartens and other organizations, 3) provide all residents to be outdoors enjoying fresh air at least once a week, 4) facilitate contact with animals if desired, 5) ensure that the residents can maintain their hobbies and interests, and 6) experience meaningful musical and cultural stimuli, as well as 7) facilitating a pleasant atmosphere during meals, 8) good practices for communication with family and next of kin, and finally 9) the NH must ensure that seasons and holidays are noticeable in the daily routines. Consequently, implementation of the JoLNH certification strategy represents introducing changes to the health professionals in the NHs.

During an implementation it is important to monitor the process [7], and the present study was done in collaboration with the municipality where the implementation took place. The researchers were independent of both those who implemented JoLNH and the municipality. The aim of this study is to illuminate the mechanisms which can lead to successful implementation, including barriers and facilitators; this study is not part of a formal evaluation of the JoLNH implementation [8]. The JoL foundation [9] conducts the JoLNH implementation including the certification process, which lasts for 1 year, during which the NHs and the municipalities undertake the necessary changes to be a certified JoLNH [2, 10, 11]. The municipalities pay a fee to the JoL foundation for the certification process as well as the annually recertification. Furthermore, the municipality employs 2–3 JoL coordinators who assist the NHs in the certification and recertification processes. The JOL certification scheme represents a systematic use of specified tools, including some teaching, guidance and support provided to the municipality, the NH management and the NH staff [9].

There is no doubt that care quality and recruitment of professional nursing competence in NHs are necessary and important [4, 12] for creating a good place to be for both residents and staff [13, 14]. A professionally evolving work environment contributes to recruitment and less turnover [1]. It is therefore vital to investigate issues related to the implementation of JoLNH authorization focusing on implementation facilitation, co-determination, and a strengthened work environment. Mostly, previous research has focused on the residents’ benefit of the JoLNH implementation [2, 10, 11, 15]. However, knowledge about the healthcare personnel’s experiences of this implementation is scarce. Therefore, the aim of this study is to explore if the Joy-of-Life-Certification-Program is adapted in accordance with the healthcare personnel’s experiences; how do the healthcare personnel perceive the JoLNH implementation, and which challenges do healthcare personnel experience with the implementation of JoLNH?

## Method

The present study was done in collaboration with the municipality where the implementation took place since mentoring an implementation process is important [7]. The researchers were independent of both those who implemented JoLNH and the municipality.

During winter 2019 healthcare personnel working in NHs who had implemented the JoLNH strategy, were invited to participate in qualitative research interviews. The NHs is in a Norwegian urban municipality. In the further, this municipality is termed the JoL-municipality. We used a retrospective approach within the qualitative paradigm to explore how the health professionals had experienced the implementation. Semi-structured individual interviews were chosen as the preferred method [16]. To get at deeper meaning of the informants’ experiences, we chose content analysis as our analysis strategy [17].

### Sample

The informants were purposively sampled in the 27 NHs in the JoL-municipality [16]. The NH management recruited the healthcare personnel and provided the researchers with a list of potential volunteered informants and their contact information. All informants were informed about the topic before the interview and volunteered to participate. The inclusion criteria were that the informants had been working in the unit before, during and after the implementation of the JoLNH strategy. The time between the implementation of JoLNH and the interview varied between three and one year. The interviews were held at the informants’ workplace during their working hours, in a room ensuring no disturbance. Two informants, with whom an interview was agreed, were absent due to illness; these were therefore not included.

### Qualitative method

The qualitative research interview is an interpersonal situation, a conversation between two partners about a theme of mutual interest [16, 17]. The interviewer attempts to verify the interpretation of the informant’s answers in the course of the interview [18, 19]. The individual interviews were held over a 6-month period and each interview lasted from 30 to 40 min. To obtain an overview over the total amount of experiences from the JoLNHs in the sample, we made sure that the informants represented different JoLNHs with one or two informants from each. The interviewers used a semi-structured interview guide so that the informants could speak more freely around the subject [16]. Focus group interviews often bring out differences of opinion, but on some topics, people can speak less freely, and the JoLNH survey contains such topics. Additional File 1 shows the interview guide developed for this study. The informants were asked question about; “How did you perceive the ongoing JoLNH certification process and the JoLNH working approach; how is /was your work in the NH influenced by the JoLNH implementation?” and “How do you think this implementation (the JoLNH certification process) works out?”. The first author (BA) has extended experience with qualitative research [20–22] and trained the two interviewers that conducted the interviews. The interviewers had successfully passed master courses in scientific methodology and qualitative research methods. They conducted a pilot interview together with the fist author (BA) who was the responsible facilitator. All interviews were audiotaped and transcribed verbatim continuously by the two interviewers to obtain saturation.

The analysis was conducted as collaborative negotiations between the authors, following Kvale’s approach to qualitative content analysis. Five approaches were used for this purpose: categorization of meaning, condensation of meaning, structuring of meaning through narratives, interpretation of meaning, and ad hoc methods for generating meaning [17]. To secure the confirmability of the material, two researchers reviewed and analyzed the interview material [19]. We systematized, condensed, and sorted the data material in preliminary categories using NVivo 12Pro. Then, we identified and highlighted meaning statements within the text, still with their original words intact. After several collaborative negotiations between the authors, we agreed on the final categories and subcategories, see Table 1. In this process, the interpretation of meaning took place in connection with the total statement before the final selection and range were made [17], the reporting follows the COREQ-checklist found in the Supplementary Material.

**Table 1:** Example of meaning units, condensation, categories, and coding

Meaning units	Condensation	Categories	Coding
Before we became a JoLNH, I think we did a lot of the same thing anyway, in everyday life when it suited us. There is a lot of tiny JoL throughout a day, even if it is not planned.	There has been a lot of joy in life through one day, even if it was not planned	The characteristics of care activities before implementations of JoLNH	Spontaneous activities

### Ethical considerations

The ethical guidelines of voluntary participation, written informed consent and the possibility of withdrawal at any point were followed. The informants were informed about the purpose and aim of the study and gave written consent to participate. All data gathered were anonymized. The Norwegian Centre for Research Data, Data Protection Services, have registered and approved the project (ref.nr. 238,331). Prior to this, an application was sent to the Regional Committee for Medical and Health Research Ethics, who declared that approval for the current project was not required according to the Norwegian Health Research Act.

## Results

### Sample characteristics

The sample (*N* = 14) comprised nine nurses (five were unit leaders), four assistant nurses, and one occupational therapist. The informants work experience in NHs ranged from 10 to 40 years, with a mean of 25 years. All informants were native Norwegians and female. Characteristics of the NH residents staying in the included NHs have been published in earlier articles related to this implementation [2, 10, 11, 15].

### Identified themes

The main identified categories were: (a) the characteristics of care activities before implementations of JoLNH, (b) how JoLNH influenced the care activities, and (c) challenges with the implementation of JoLNH. The findings are further elaborated with statements from the informants. The informants are numbered in parentheses at the end of each statement.

### The characteristics of care activities before implementations of JoLNH

The informants gave several examples of different activities they offered to the residents to make their days meaningful, before the NH implemented the JoLNH strategy. However, the informants said the organization and planning of these activities were spontaneous, and happened by coincidence, depended on the healthcare personnel at work. One informant said they used song and music to bring forward memories and give recognition, another told that they read aloud from the newspaper during breakfast, and a third explained that they had joyful moments only by being together with the residents, laughing, walking and such like. One informant expressed the spontaneous activities like this:

*There has been a lot of joy in life through one day, even if it was not planned* [16]*.*

Even though the care was characterized by spontaneous activities, the informants explained that they used life history mapping to get an overview over their residents’ prior interests and activities. However, before the implementation of JoLNH informants explained that they (the care personnel), unconsciously might have focused most on residents that could speak out about their needs and less on the residents who did not demand anything:

It was perhaps the case that the residents who were most fond of talking received the most – it might feel more naturally to sit down with them [22]

### How JoLNH influenced the care activities

The second main category is supported by data on how the informants described that the JoLNH influenced the care. The informants talked about how the JoLNH lead to better planning and implementing systems for the care activities, the strategy increased their awareness on dignity, and entailed more involvement of the individual residents and their relatives, this became particularly noticeable in relation to end of life care. The informants stated that one of the requirements of implementing the JoLNH strategy was more documentation. The documentation lead to more systematic work with joy of life care activities. One informant explained how the implementation of JoLNH contributed to placing the care activities into a system. Another said that the staff had become more aware of the individual residents’ needs and worked more systematic to tailor joy of life activities to different residents’ needs. Several informants said they experienced the residents as more satisfied after the implementation.

With the JoLNH – we map all the residents about their background, what they like to do, and what they don't like to do [12]

It is positive, now everyone (the residents) get an activity program that is adapted to their needs, it is more systematic [13]

Several informants said they experienced that the residents were more satisfied after the implementation of JoLNH. They further emphasized that the residents deserved to have care activities adapted to their situation and be treated with dignity*.* The JoLNH also made the staff more committed to ensure that the care activities were targeted to the individual residents’ needs and executed in accordance with the plan. For some residents, the care activities could be that the care personnel sang along with the resident, had a small chat, took the resident outside to the porch for fresh air, or used music-based environmental treatment. To make sure that the residents’ individual needs were considered, the informants used each resident’s life history to plan the activities.

We create a monthly plan and then we evaluate afterwards. We should describe the resident's experiences and activities if there has been nothing positive have happened then we must report that [7]

Another positive experience related to the JoLNH implementation was that the healthcare personnel became more conscious about seeing each resident as an individual person and involving relatives. One informant emphasized the JoLNH criteria as helpful in communicating with the relatives and explained that the documentation of care activities could be printed and given to the relatives. This documentation showed what their mother or father had been doing that day and was greatly appreciated by in the relatives. Another informant told a story about an old female resident that loved flowers, and how the JoLNH strategy had made the staff more aware of this woman’s needs by letting her go outside to pick flowers.

To see the joy in the eyes of the resident then I feel we have succeeded to engage them in activities which we did not before [7]*.*

### Challenges with the implementation of JoLNH

Informants commented that even though they saw the documentation as positive, it was also challenging and difficult to perform. One informant said that the documentation was too much; it was time-consuming and difficult to remember in addition to all the other tasks they had to carry out. One informant explained that fewer specific requirements for how to document could make the documentation less time-consuming and easier to do.

All this documentation is a working task that has come in addition to everyday working tasks. I understand that we must document the experience, but objectivity is important [10]

Informants said that the JoLNH demanded for more resources if they should fulfil the JoLNH criteria and perform the JoLNH care activities. Some told that they had special JoLNH personnel that was responsible for the activities. Even though several of the informants stated that they used to carry out similar JoLNH activities before the implementation of JoLNH, they thought about the strategy as something that demanded extra resources. One informant told that some days, it could be difficult to let all residents go outside and get fresh air. Another informant experienced some JoLNH arrangements as challenging because the unit was empty, and someone needed to stay and take care of the residents who are uneasy and not able to participate. In such situations, the informant said she often had to sacrifice the lunchbreak to make it work. Others said it was important to get people involved and to understand how important JoLNH was, and that it did not have to be so hard and time-consuming.

We struggle with having enough time, we know how the staffing situation is, there are many tasks and many times, we feel we have too little time [2]

Informants also commented that the JoLNH activities were not suited to all residents living in NHs today, because the residents are frailer than some years ago. When the residents are sicker, the informants explained that it was more difficult to develop activities adapted to their individual situation. One said the clue was to think simple. Some residents enjoyed the activities while others had opposite reactions, they could become restless and insecure. Several informants said that residents with dementia perhaps had less benefit from JoLNH activities because they needed an environment that was safe, calm, and predictable.

I feel that those who are JoLNH responsible, they run around with noise and sound and events, but here in the NH 9 out of 10 residents have dementia and running around is not JoLNH for them [9]

## Discussion

In this study, we have explored the implementation of the JoLNH authorization through two research questions; how is JoLNH perceived by the healthcare personnel, and which challenges healthcare personnel experience with the implementation of JoLNH?

### Positive outcomes of implementation of JoLNH

To sum up, most informants stated that many JoL activities were present also before the implementation of JoLNH. However, it seemed like the activities before the implementation were more spontaneous then after the implementation.

Before implementing the JoLNH, the healthcare personnel perceive NH care as spontaneous care activities. Characteristically, in this study several informants describe positive outcomes for the residents resulting from the implementation of JoLNH. Among others, it seems that the implementation supports more focus on dignity for residents in NHs. Even if the residents are in the last phase of their life nursing interventions suitable for the resident’s situation are promoted. Both dignity and respect in the last phase of life were present before the implementation of JoLNH. The informants describe that they feel committed, and when it is systemized it is easier to customize the activities into everyday life. When the benefits of changes are in focus, the motivation to change behavior increases [23–26]. When the informants talk about quality of care and more planned activities, many of them emphasize the benefits for the residents. Some of the informants spoke well about the implementation concerning the quality of the care, as one stated “*to see the joy in the eyes of the resident then I feel we have succeeded”.* Earlier research has indicated that the relationships with the residents and the quality of the care strongly influence on the health care personal’s experience of coping [1, 27]. When involving relatives, the implementation of JoLNH has positive influences. The informants state that they discuss activities and nursing interventions with the relatives, which makes it easier to involve the relatives better.

Some informants shared their concern for the more fragile resident group in NHs, and that the JoLNH strategy may not be suited for this group, while other informants stated that it must be possible to have activities that take the resident’s condition in consideration. Some informants’ states that fragile residents will favor of the implementation and some informants’ states that fragile residents will not benefit of the implementation, so it seems like the influence of the implementation of the fragile resident group is uncertain.

### How is the implementation experienced – challenges and benefits

When summing up, it seems that the informants formed two different groups related to the challenges: (i) those who were positive to the documentation requirements, and (ii) those who experienced resistance to the documentation requirement.

Informants affirmed that some activities were provided for the residents also before the implementation of JoLNH. Many of these previous activities were like the activities promoted by the JoLNH program. The main difference between before and after the implementation of JoLNH, seems to be the documentation and systemization of activities. To be able to ensure that every resident gets custom activities, systematics and documentation are important [28, 29]. When describing the challenges with JoLNH many informants mentioned the obligation of documentation of performed activities, and that this documentation should be done in a specific way; hence, some healthcare personnel may miss being spontaneous. The informants experienced the documentation requirements differently, some were positive and saw the benefit of better documentation and more systematics while others stated that it was too much to document and that the documentation requirements were too complicated. One informant also stated that she had become more positive to the documentation requirements over time, when she discovered that the systematic inspection of the residents and their needs were more thorough after implementation of JoLNH. That inspection lead to discovering of more needs among the residents and additional nursing interventions improving residents’ well-being. So even if the informants experienced the documentation requirements as a load in the beginning it is possible to see the benefits of more systematic documentation after a while.

Most of the JOL implementation was done without supplying any extra resources and may be an explanation to why several informants expressed resistance towards the implementation of the documentation requirements. If it is expected that the staff is going to increase their workload, and in the same period facilitate the implementation, this may prove difficult to accept. The JoLNH responsible in the ward, was mentioned by some informants; they seemed to be used as a change agent. To perform a successful change the use of change agents or key personnel is important [30].

However, to be able to alter this resistance it is important to identify the causes for the resistance, which in this case seems to be a feeling of being overloaded with work. Resistance may occur after an implementation of changes if this lead to increased workload [31, 32]. To overcome this it might be useful to introduce some training programs to make the health care personnel more motivated and ready for the change [24, 33, 34]. To prepare the health care personnel to be able to change and to have resources to change is a management responsibility [1, 35]. The implementation does not only relate to change in the documentation system but does also connect to activities and nursing interventions. When health care personnel experience a heavy workload the importance of experiencing control over the situation is important [3, 36, 37], as one informant stated*,” you don’t have to tidy up right away”*. This informant describes that she takes control over the situation by making priorities, while another informant feels overwhelmed and *“have to sacrifice my lunchbreak”.* This clearly shows that related to this change there are at least two different ways to react.

Previous research has shown that health care personnel experience commitment and positive energy related to care quality [1]. When connecting this change to better outcomes for the residents, it increases health care personnel’s ability and desire to contribute positively to this change; one of the present informants underlined the importance of getting the staff involved and help them to understand the importance of implementing JoLNH to increase the care quality. A sense of co-determination and possibilities for professional updates for healthcare personnel are included in the JoLNH strategy [9]. To use this opportunity to both strengthen the quality of care and make it possible for the healthcare personnel to increase their professional satisfaction in their work will be important to influence on the health care workers’ intention to change [38]. Making changes to increase quality of care in NH’s is positive both for the residents and for the healthcare personnel, and one way to succeed seems to be to implement the JoLNH strategy.

### Strength and limitations

This study focuses on the user’s perspective in an implementation process, representing an important perceptive which previously has been given lesser attention. This study could be the basis to change the JoLNH training program for the healthcare staff while focusing on the described problems with the implementation process in a more detailed way.

The present findings were translated from Norwegian to English; when translating data, it is always a risk to misunderstand and lose some of the original content. Dependability and confirmability are major factors in understanding the implications of this study, and considerably effort was dedicated to examining these issues. Content analysis was used to identify similarities, differences, and patterns in the experiences of informants, and conclusions were deduced from the collected material without a predetermined hypothesis. This is a qualitative interview study, so it is the informant’s own experiences, which is the basis for the results. This means that there is no access to the daily life in the NH, something that would be acquired through an observation study. When we chose individual interviews rather than focus group interviews, we obtained the individual informant’s experiences. By choosing a focus group interview, we could have been able to establish differences of opinions between the informants, but the informants could also exercise control over each other’s statements and not spoken so freely. All informants were female, which is a limitation. Including informants of both genders could have portrayed a broader picture of the experiences of the JoLNH implementation. However, 85% of the healthcare personnel employed in Norway are female [39] so our sample seems to be characteristic for the total population. A more balanced group of informants with both genders may have given the findings other aspects of the implementation process.

## Conclusion

Most informants in this study reported positive experiences with the implementation of JoL strategy and perceived several benefits related to the care quality because of the implementation. The JoL strategy made the healthcare professionals more conscious of planning and implementing systems for the care activities and entailed more involvement of the individual residents and their relatives. Nevertheless, the healthcare professionals also experienced the implementation as challenging, especially the demanded documentation and systemization of activities without additional resources. There was disagreement among the informants about the most fragile residents benefit of the implementation.

In future research, challenges related to an implementation of a new strategy or care could be more extensively examined utilizing focus groups with healthcare personnel.

## Data Availability

The datasets used and/or analyzed during the current study available from the corresponding author on reasonable request.

## Abbreviations

NH: Nursing homes
QoL: Quality of life
JoLNH: Joy of Life Nursing Homes
JoL: Joy of Life
