# Correction to: “Joy of life” in nursing homes. Healthcare personnel experiences of the implementation of the national strategy. A qualitative study with content analysis of interviews

**DOI:** 10.1186/s12913-021-06875-6

**Published:** 2021-09-29

**Authors:** Beate André, Kjersti Grønning, Frode F. Jacobsen, Gørill Haugan

**Affiliations:** 1 Department of Public Health and Nursing, Norwegian University of Science and Technology (NTNU), 7491 Trondheim, Norway; 2 NTNU Center for Health Promotion Research, 7491 Trondheim, Norway; 3 Centre for Care Research, Western Norway University of Applied Sciences, Bergen, Norway; 4 Institute for Health and Care Sciences, Western Norway University of Applied Sciences, Bergen, Norway; 5 VID Specialized University, Bergen, Norway

**Correction to: BMC Health Serv Res 21, 771 (2021)**

**https://doi.org/10.1186/s12913-021-06801-w**

Following publication of the original article [1], the numbers after the informants’ quotes should be the informants number, not the citation number.

The original article [1] has been updated.
